# Characterization of Alzheimer's disease‐like neuropathology in Duchenne's muscular dystrophy using the DBA/2J *mdx* mouse model

**DOI:** 10.1002/2211-5463.13317

**Published:** 2021-11-11

**Authors:** Grant C. Hayward, Daniela Caceres, Emily N. Copeland, Bradley J. Baranowski, Ahmad Mohammad, Kennedy C. Whitley, Val A. Fajardo, Rebecca E. K. MacPherson

**Affiliations:** ^1^ Faculty of Medicine University of Ottawa Canada; ^2^ Faculty of Medicine University of del Rosario Bogota Colombia; ^3^ Department of Kinesiology Brock University St. Catharines Canada; ^4^ Department of Health Sciences Brock University St. Catharines Canada

**Keywords:** Alzheimer's disease, amyloid, brain, cognition, Duchenne muscular dystrophy, muscular dystrophy

## Abstract

Duchenne muscular dystrophy (DMD) is a progressive muscle wasting disorder caused by a mutation in the dystrophin gene. In addition to muscle pathology, some patients with DMD will exhibit cognitive impairments with severity being linked to age and type of genetic mutation. Likewise, some studies have shown that *mdx* mice display impairments in spatial memory compared with wild‐type (WT) controls, while others have not observed any such effect. Most studies have utilized the traditional C57BL/10 (C57) *mdx* mouse, which exhibits a mild disease phenotype. Recently, the DBA/2J (D2) *mdx* mouse has emerged as a more severe and perhaps clinically relevant DMD model; however, studies examining cognitive function in these mice are limited. Thus, in this study we examined cognitive function in age‐matched C57 and D2 *mdx* mice along with their respective WT controls. Our findings show that 8‐ to 12‐week‐old C57 *mdx* mice did not display any differences in exploration time when challenged with a novel object recognition test. Conversely, age‐matched D2 *mdx* mice spent less time exploring objects in total as a well as less time exploring the novel object, suggestive of impaired recognition memory. Biochemical analyses of the D2 *mdx* brain revealed higher soluble amyloid precursor protein β (APPβ) and APP in the prefrontal cortex of *mdx* mice compared with WT, and lower soluble APPα in the hippocampus, suggestive of a shift towards amyloidogenesis and a similar pathogenesis to Alzheimer's disease. Furthermore, our study demonstrates the utility of the D2 *mdx* model in studying cognitive impairment.

## Graphical abstract

Age‐matched D2 *mdx* mice spend less time exploring the novel object compared with wild‐type (WT), suggestive of impaired recognition memory. Biochemical analyses of the D2 *mdx* brain revealed higher soluble amyloid precursor protein β (APPβ) and APP in the prefrontal cortex of *mdx* mice compared with WT, and lower soluble APPα in the hippocampus, suggestive of a shift towards amyloidogenesis.

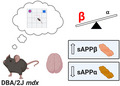

**Abbreviations**
AD: Alzheimer's disease
APP: amyloid precursor protein
Aβ: amyloid‐β
BACE1: β‐site APP cleaving enzyme 1
BCA: bicinchoninic acid
C57: C57BL/10
D2: DBA/2J
DMD: Duchenne muscular dystrophy
DR: discrimination ratio
NORT: novel object recognition test
sAPP: soluble amyloid precursor protein
TBST: tris‐buffered saline/0.1% Tween 20
WT: wild‐type

Duchenne muscular dystrophy (DMD) is a progressive muscle wasting disorder caused by a mutation in the dystrophin gene [1]. DMD affects approximately one in 3500 male births with disease onset occurring around 3–5 years of age. Clinical features of DMD include progressive muscle wasting, impairment of motor development and loss of ambulation, and eventually premature death most commonly due to cardiorespiratory complications [2]. Although most studies focus on the role of skeletal and cardiac muscle in DMD pathogenesis, small amounts of dystrophin have also been found in the brain, specifically in memory‐associated brain regions such as the hippocampus and cortex [3, 4, 5]. Importantly, several dystrophin isoforms have been identified throughout the development of the human brain, including isoforms found in the cortex and the hippocampus [6] – highlighting an important role for dystrophin in brain function.

Given that dystrophin is expressed in neurons, it likely plays an important functional role in the central nervous system. Boys with DMD exhibit varying degrees of cognitive impairment (for review, see [7]). Specifically, 30% of boys with DMD show cognitive impairment with an intelligence quotient (IQ) < 70 [8] and 40% with reading deficits [9, 10]. However, other studies showed that boys with DMD had intact language skills [11], or had comparable IQ levels to the norms for their age [12]. Indeed, age can account for this variability as younger boys with DMD seem to have lower IQ scores compared with their age‐matched healthy controls, whereas a normalization in IQ has been observed in older boys with DMD [12, 13, 14, 15]. Further, there are over 7000 mutations in the dystrophin gene known to cause DMD [16], which combined with the several isoforms of dystrophin found in the brain can also contribute to the varying degree of cognitive impairment in boys with DMD. For example, patients missing all isoforms due to mutations occurring in the distal part of the dystrophin gene have the lowest IQ scores, whereas those only missing the Dp427 full‐length isoform have the highest IQ scores [17]. Moreover, patients missing a shorter Dp140 isoform appear to have the most impaired cognitive function and profound reductions in grey matter volume and altered white matter microstructure compared with age‐matched healthy controls [18, 19, 20]. Collectively, these data indicate that some boys with DMD display cognitive deficits; however, the underlying cellular mechanisms are not well understood.

Recently, Salam et al. [21] reported that patients with DMD had significantly higher levels of serum amyloid‐β (Aβ) 42 compared with controls. Furthermore, they show a negative correlation between Aβ42 levels (21.9 ± 6.7 DMD vs 12.13 ± 4.5 Controls) and IQ (74.8 ± 9.3 DMD vs 95.4 ± 10 controls; *r* = −0.74 and *P* < 0.05). The accumulation of Aβ is the main hallmark of neurodegeneration that occurs in Alzheimer's disease (AD) [22, 23, 24, 25]. These peptides originate from a type I transmembrane protein, known as amyloid precursor protein (APP), which can be cleaved and processed through two competing processes. The nonamyloidogenic pathway first produces a soluble APPα fragment and avoids the production of Aβ peptides. In contrast, the amyloidogenic pathway involves APP first being cleaved by β‐site APP cleaving enzyme 1 (BACE1) to form a soluble APPβ fragment, before being cleaved further to form Aβ [26, 27]. Although human studies suggest accumulation of Aβ in the serum of patients with DMD, whether this is the result of brain changes in markers of amyloidogenesis requires further investigation. Despite the neuropathogenesis of this disease being elusive, a role of synaptic dysfunction has been hypothesized [7, 28]. Given limitations in access to brain samples from patients with DMD, further characterization of brain markers involved in these two processes, using the *mdx* mouse model of DMD, is required.

The *mdx* mouse is the traditional model for DMD, and studies in these mice have demonstrated impairments in passive avoidance learning [29], retention in a T‐maze and in long‐term consolidation [30], object recognition and spatial memory [31]. However, as with human patients with DMD, there is some discrepancy in the field and a recent study did not find any differences in cognitive flexibility in *mdx* mice compared with wild‐type (WT) controls [32]. These previous studies have all utilized the *mdx* mouse on a C57BL/10 (C57) background, which despite being the most commonly used *mdx* model is known to have a mild dystrophic phenotype compared with human DMD. Conversely, *mdx* mice backcrossed onto a DBA/2J (D2) background have been shown to have earlier onset and worsened muscle pathology, compared with the C57 *mdx* mice, making it a more viable model to study muscular dystrophy [33, 34, 35]. Understandable with its recent emergence, studies examining cognitive function in the D2 *mdx* mice are limited. Thus, the focus of this paper is to examine cognitive function in these mice to gain a better understanding of the neuropathology associated with DMD in memory‐specific brain regions (the hippocampus and prefrontal cortex). We hypothesize that the D2 *mdx* mice will show impairments in cognitive function and elevated markers of amyloidogenesis and synaptic dysfunction.

## Materials and methods

### Materials

Molecular weight marker, reagents and nitrocellulose membranes for SDS/PAGE were acquired from Bio‐Rad (Mississauga, ON, USA) and Millipore Sigma (GE10600002, Burlington, MA, USA). Antibodies against soluble amyloid precursor protein β (sAPPβ; 1 : 500; BioLegend, San Diego, CA, USA, cat# SIG‐39138), sAPPα (1 : 500, BioLegend cat#SIG39139), APP (1 : 500 BioLegend, cat#SIG039152), BACE1 (1 : 500; Cell Signaling, Whitby, Canada, cat#5606P), synaptophysin, acquired from Cell Signaling Technology (concentrations cat#5461); PSD‐95, acquired from Santa Cruz Biotechnology (concentrations cat# sc‐32290); Homer1, acquired from Santa Cruz Biotechnology (concentrations cat#sc‐136358). Horseradish peroxidase anti‐mouse and anti‐rabbit secondary antibodies were purchased from Jackson ImmunoResearch Laboratories, West Grove, PA, USA (Donkey anti‐rabbit IgG (H + L) 711‐035‐152, Goat anti‐mouse IgG (H + L) 115‐035‐003 Jackson ImmunoResearch) and Western Lightning Plus Enhanced Chemiluminescence from Perkin Elmer (Guelph, Canada, cat#105001EA).

### Animals and design

Male D2 *mdx* (8–12 weeks of age, *n* = 12; stock number = 013141) and male WT D2 mice (*n* = 12; stock number = 000671) as well as C57*mdx* (*n* = 12; stock number = 001801) and C57 WT (*n* = 12; stock number = 000476) were purchased from Jackson Laboratories. Mice were housed in standard 12 : 12‐h light : dark cycles and were allowed access to standard rodent chow and water ad libitum. All mice were euthanized via exsanguination under general anaesthetic (5% vaporized isoflurane), and the left and right prefrontal cortex and hippocampus were quickly dissected and snap‐frozen in liquid nitrogen and stored in a −80 °C freezer until western blot analyses. All animal protocols were approved by the Brock University Animal Care Committee (AUP 17‐06‐03) and were in compliance with the Canadian Council on Animal Care.

### Novel object recognition test

The novel object recognition test (NORT) was performed in an arena consisting of four equal sized, open top boxes. Exploration was recorded with a video camera, secured above the apparatus. Prior to testing, pilot work was done to test for inherent object preference/aversion. No intrinsic bias towards the objects was observed. Further, pilot work demonstrated no intrinsic preference to specific locations in the arenas. Testing was performed in three distinct stages: prehabituation, habituation and testing as previously described [36, 37, 38]. Each stage was performed at the same time of day. As an additional precaution to avoid potential arena bias between the 4 arenas, a set of each experimental group was tested in each arena. During prehabituation, mice were placed in the arenas and allowed to freely explore for 10 min. The following day in the habituation stage, mice were placed in the box with two identical objects placed in the bottom corners and were left to explore for 10 min. Objects were chosen based on difference in shape, to ensure novelty in the trials and similarity in size to the mice. Following habituation, mice were returned to their home cages for delay period of 60 min. Mice were then placed back in their respective boxes with one familiar object and one novel object, again exploring for 10 min. Object exploration time was defined as the time the mouse sniffed the object or touched the object while looking at it (i.e. when the distance between the nose and the object was <2 cm). Any sitting or standing on the object was not included (unless the mouse sniffs the object it has climbed on). Exploration index (or per cent of total investigation time) was calculated as the per cent of the time spent investigating the novel object divided by the sum of the times spent with the novel and familiar objects. Discrimination testing was performed on all objects prior to habituation and testing to remove object bias. Discrimination ratio (DR) was calculated as follows: DR = (Time (object 1) – Time (object 2))/Total time. In addition to object exploration time, total movement time was analysed as an indicator of mobility.

### Western blotting

Prefrontal cortex and hippocampus brain tissue (either left or right) were homogenized (FastPrep, MP Biomedicals, Santa Ana, CA, USA) in 20 volumes of NP40 Cell Lysis Buffer (Life Technologies, Burlington, Canada; CAT# FNN0021) supplemented with 34 μL phenylmethylsulfonyl fluoride (Sigma, Oakville, ON, USA, P7626) and 50 μL protease inhibitor cocktail (Sigma P2714). Samples were then centrifuged for 15 min at 10 000 ***g*** and 4 °C, and supernatants were collected. Homogenate protein concentrations were determined using bicinchoninic acid (BCA) quantification assay [39]. Total protein concentrations were equalized, and western blot samples were prepared using 2× Laemmli buffer. 20 µg of protein was loaded into each well on 10% SDS/PAGE and separated for 1.5 h at 120V. Following this, proteins were wet transferred to nitrocellulose membranes for 1 h at 100V on ice. Membranes were blocked for 1 h at room temperature in 5% nonfat dry milk‐ tris‐buffered saline/0.1% Tween 20 (TBST). Membranes were then incubated in the appropriate primary antibody, diluted 1 : 500 in 5% BSA‐TBST overnight with gentle agitation at 4 °C. The following day, membranes were rinsed 3× with TBST and incubated at room temperature for 1 h in horseradish peroxidase secondary antibodies diluted 1 : 2000 in 1% nonfat dry milk‐TBST. Membranes were washed (3 × 5 min in TBST). Protein bands were imaged using enhanced chemiluminescence (Western lightning Plus‐ELC; PerkinElmer, 105001EA) and ChemiDoc Imaging System (Bio‐Rad). Western blot analysis was used to examine proteins of amyloidogenesis (sAPPα, sAPPβ, BACE1, APP), and synaptic markers (synaptophysin, PSD‐95, and Homer1) were quantified. A representative Ponceau stain was analysed for each membrane to ensure equal loading (< 10% variability across the membrane).

### Statistical analysis

Novel object recognition test and western blot results of WT vs *mdx* mice were compared using an unpaired Student's *t*‐test. Normality was assessed using a Shapiro–Wilk test. All data were presented as mean ± SEM, with significance reported as *P* ≤ 0.05, and values approaching significance were reported as *P* = 0.06–0.10.

## Results and Discussion

### NORT test

**Fig. 1:**
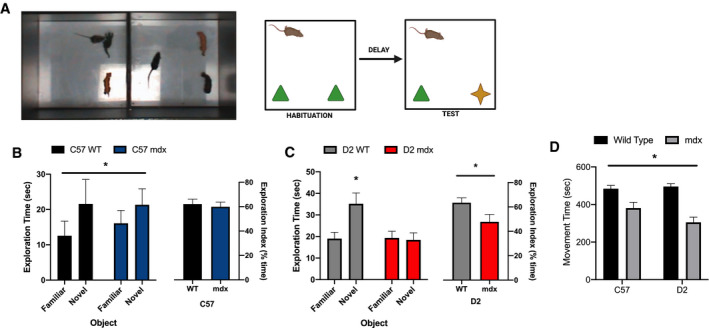
NORT. (A) Representative images of one arena with two objects, one familiar and one novel (left panel), and test design (right panel). (B) C57 WT and mdx exploration time of familiar and novel object in recorded in seconds and exploration index (%). (C) D2 WT and *mdx* exploration time of familiar and novel object in recorded in seconds and exploration index (%). (D) Total movement time in seconds throughout the NORT. Data expressed as mean ± SEM with *n* = 12/group. Significance is set to *P* ≤ 0.05 indicated by *.

Novel object recognition test was used to examine memory performance as it relates to nonspatial learning of object identity and relies on a variety of brain regions including the hippocampus (Fig. 1A). Briefly, mice with a memory of the previously encountered objects will prefer investigating the novel object. During the habituation phase of the NORT task, both experimental groups spent equal amounts of time with the two identical objects, demonstrating that both genotypes were equally interested in both objects (data not shown). We first examined memory performance in the C57 WT and C57 *mdx* mice, and our results showed a significant main effect of the novel object, where both C57 WT and C57 *mdx* mice explored the novel object more than the familiar object (*P* < 0.05; Fig. 1B). In contrast, we found a significant interaction between the novel object and *mdx* genotype when examining mice on the DBA/2J background (*P* < 0.05). Post hoc analyses revealed that only the D2 WT mice explored the novel object, whereas the D2 *mdx* mice did not (Fig. 1C). Previous work demonstrating reduced novel object exploration time in *mdx* mice attributed this with a heightened fear and anxiety‐like behaviour when challenged with NORT36. While this could be the case, it does not determine if the reduction in novel object investigation is simply due to impaired motor function in the D2 *mdx* mice. To examine this, we investigated total movement time throughout the test (Fig. 1D). A main effect of *mdx* was demonstrated, revealing that both C57 and D2 *mdx* mice had a lower movement time compared with the WT mice. There was no difference in movement time between the C57 *mdx* and D2 *mdx* mice. These data paired with the reduction in novel object exploration in the D2 *mdx* mice demonstrate a true cognitive deficit in this strain. Furthermore, when calculated as a percentage of total exploration time, the mdx mice had a lower exploration index compared with the WT mice (*P* < 0.05; Fig. 1C). This suggests that D2 *mdx* mice have a larger impairment in recognition memory when compared to age‐matched C57 *mdx* mice. This adds to previous work comparing muscle pathology in D2 and C57 *mdx* mice [40], where the DBA/2J background presents a more severe and earlier onset of the disease phenotype (i.e. inflammation and fibrosis) and muscle weakness with a potential role for overactive TGF‐beta [33, 34, 35, 41].

Despite showing that *mdx* mice had impaired recognition memory here, changes in cognition have been inconsistent across the literature. Specifically, Chaussenot et al. [42] found no differences in spatial working memory with C57 mice using the water and radial‐arm maze. Furthermore, Valliend et al. [31] found that *mdx* mice had intact short‐term object recognition while demonstrating impaired long‐term object recognition memory and spatial memory. It is important to note that on top of alterations in cognitive capacity in *mdx* models that there are likely multiple behavioural changes that should be evaluated. Given the variability in the field thus far in regard to types of testing and results, it would be of great benefit to develop a set of standardized tests to evaluate *mdx* mice across laboratories. Operant conditioning testing is one such method that can provide novel insight into alterations in behaviour in *mdx* models. For example, in an attempt to systematically assess operant learning in the C57 *mdx* model, Lewon et al. investigated multiple operant conditioning modalities, assessing both learning and memory, between *mdx* and WT mice [43]. In this study, *mdx* mice showed enhanced learning when food was the motivator, but impaired escape/avoidance learning, relative to WT mice. Thus, similar studies in the D2 *mdx* mouse should also be conducted.

### Amyloidogenic markers

**Fig. 2:**
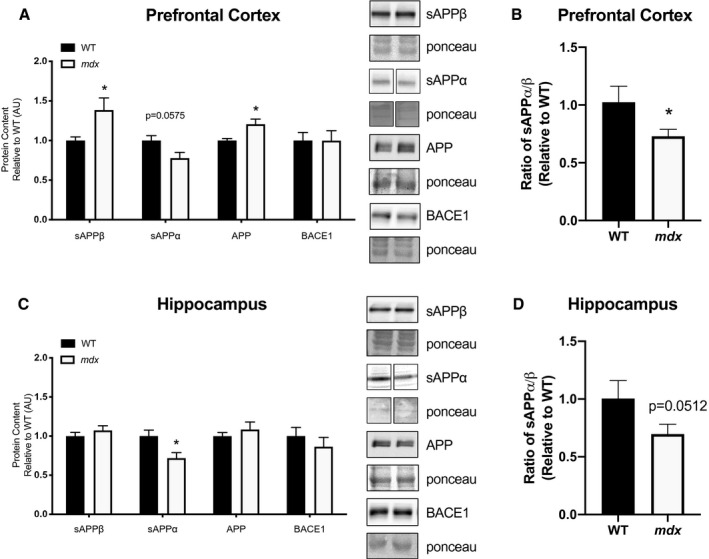
APP processing markers, and BACE1 content (A) sAPPβ, sAPPα, APP and BACE1 prefrontal cortex; (B) sAPPα/β ratio prefrontal cortex; (C) sAPPβ, sAPPα, APP and BACE1 hippocampus; (D) sAPPα/β ratio hippocampus. All graphs accompanied by representative blots. Data analysed using an unpaired Student's *t*‐test. *mdx* = muscular dystrophy mouse model. All values were made relative to the control group (WT). Data expressed as mean ± SEM with *n* = 12/group. Significance is set to *P* ≤ 0.05 indicated by *.

Amyloidogenic markers were characterized in the *mdx* brains compared with WT. Given that cognitive impairment was only observed in the D2 *mdx* mice, we focused our analyses on these mice and not the C57 *mdx* mice. The sAPPα fragment is a product of the nonamyloidogenic pathway, whereas the sAPPβ fragment is a product of the amyloidogenic pathway. Therefore, reduced sAPPα : sAPPβ ratio is indicative of a shift towards the amyloidogenic pathway. In the prefrontal cortex, sAPPβ was significantly higher in the *mdx* group (*P* < 0.05), while sAPPα was decreased; however, this value was not statistically significant (*P* = 0.0575; Fig. 2A). Furthermore, in the prefrontal cortex, *mdx* mice had higher total APP (*P* < 0.05), with no changes in BACE1 content (Fig. 2A). Examination of the ratio of sAPPα to sAPPβ revealed a lower ratio in *mdx* prefrontal cortex (*P* < 0.05; Fig. 2B). In the hippocampus, sAPPα was lower in the *mdx* group compared with the WT (*P* < 0.05), with no changes in sAPPβ (Fig. 2C); however, the ratio of sAPPα to sAPPβ was lower in *mdx* (*P* = 0.05; Fig. 2D). No differences were found for APP and BACE1 content in the hippocampus.

For the first time, we show both a reduction of sAPPα and an increase in sAPPβ in memory‐specific brain regions of *mdx* mice. Altogether, these data suggest that in both the prefrontal cortex and hippocampus, there is a shift towards the amyloidogenic pathway in *mdx* mice, which we believe can contribute to the memory impairments observed in these mice (Fig. 1). Decreased cerebrospinal fluid levels of sAPPα have been observed in patients with AD [44], along with lower levels correlating with cognitive impairment in both patients with AD and Fischer‐344 rats [45, 46]. The role of sAPPα is thought to increase synaptic function and neuroprotective properties [47]. In this study, we also report an increase in the sAPPβ fragment, a product of the amyloidogenic pathway. In patients with AD, sAPPβ has previously been correlated to Aβ levels, the neural damage marker that accumulates in AD and has been shown to be elevated in patients with DMD [21, 48, 49]. Researchers have suggested that sAPPβ is significantly less potent and therefore less neuroprotective than sAPPα [50], with some even suggesting that sAPPβ can exhibit neurotoxicity by stimulating microglia and producing proinflammatory cytokines [51]. This could indicate that an increase in sAPPβ could be damaging independently, indifferent of changes in Aβ levels. A limitation in the current investigation is the lack of a measurement of Aβ peptides. The downstream consequences of APP processing through the amyloidogenic cascade include the increased production of Aβ, which has been shown to disrupt synapse formation and mitochondrial function, impair memory, and eventually lead to neuronal loss [52, 53, 54]. Therefore, DMD is not only associated with elevated circulating Aβ [21], but brain dystrophin loss is associated with a shift towards the amyloidogenic pathway in memory‐specific brain regions of *mdx* mice. This provides further explanation for cognitive dysfunction and memory impairments seen in patients with DMD.

We also measured BACE1 content, which is largely thought to be the rate‐limiting enzyme involved in the amyloidogenic cascade. Although we report no changes in total BACE1 content, it is possible that BACE1 enzymatic activity differs between *mdx* and WT. We recommend future studies in our laboratories and others examine overall BACE activity and seek to understand mechanistically the potential relationship between this enzyme and dystrophin.

### Synaptic markers

**Fig. 3:**
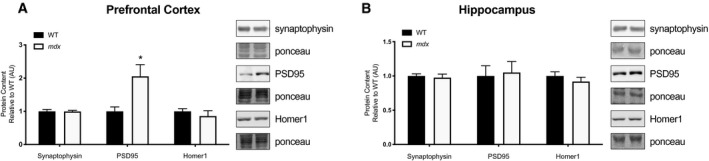
Synaptic markers. (A) synaptophysin, PSD‐95, and Homer1 prefrontal cortex; (B) synaptophysin, PSD‐95, and Homer1 hippocampus; all graphs accompanied by representative blots. Data analysed using an unpaired Student's *t*‐test. *mdx* = muscular dystrophy mouse model. All values were made relative to the control group (WT). Data expressed as mean ± SEM with *n* = 12/group. Significance is set to *P* ≤ 0.05 indicated by *.

Synaptic markers, synaptophysin, PSD‐95 and Homer1 were measured in the prefrontal cortex and hippocampus. In the prefrontal cortex, *mdx* mice had significantly higher PSD‐95 content compared with WT mice (*P* < 0.05; Fig. 3A). There were no other synaptic marker changes in either brain regions (Fig. 3A,B). Comim et al. [55] report a similar finding where brain tissue of *mdx* mice had increases in both PSD‐95 and synaptophysin. Although our results are comparable to this study, the mechanism behind this increase in postsynaptic markers remains elusive. Comim et al. hypothesized that the increase in PSD‐95 could be due to increases in excitotoxicity, subsequent glutamate receptor activation, and eventual downstream changes in postsynaptic structures. Future research should aim to further elucidate this mechanism and examine functional changes to synaptic signalling.

## Conclusion

The purpose of this study was to characterize brain changes in *mdx* mice, in attempts to further understand cognitive dysfunction in DMD. Consistent with some previous studies, we did not observe any deficits in recognition memory in C57 *mdx* mice compared with their respective controls. In contrast, age‐matched D2 *mdx* mice – a more severe model of DMD – displayed a significant reduction in exploration time. In these mice, we report for the first time, a shift towards amyloidogenesis in memory‐specific brain regions, suggesting pathogenesis similar to AD. Thus, future studies may utilize this D2 *mdx* model to inform on other potential mechanisms or therapeutic strategies that could potentially apply to human DMD.

## Conflict of interest

The authors declare no conflict of interest.

## Author contributions

GCH, VAF and REKM conceived and designed the project. GCH, DC, ENC, BJB and AM acquired data. GCH, DC, ENC, BJB, AM and VAF analysed and interpreted the data. KCW was responsible for animal husbandry. GCH and DC wrote the manuscript. All authors contributed to editing. VAF and REKM provided funding.

## Data Availability

The data that support the findings of this study are available from the corresponding author (rmacpherson@brocku.ca) upon reasonable request.
